# Embryo biopsies for genomic selection in tropical dairy cattle

**DOI:** 10.1590/1984-3143-AR2023-0064

**Published:** 2023-07-24

**Authors:** Clara Slade Oliveira, Luiz Sergio Almeida Camargo, Marcos Vinicius Gualberto Barbosa da Silva, Naiara Zoccal Saraiva, Carolina Capobiango Quintão, Marco Antonio Machado

**Affiliations:** 1 Embrapa Gado de Leite, Juiz de Fora, MG, Brasil

**Keywords:** genome selection, bovine, embryo biopsy, dairy breeding programs

## Abstract

Genomic selection has transformed the livestock industry, enabling early-life selection of animals. Biopsy sampling of pre-implantation embryos has been described since 1968. However, it was only after 2010, with the advancement of molecular biology techniques such as whole genomic amplification and SNP Chips, that next-generation sequencing became commercially available for bovine embryos. It is now possible to make decisions about which embryos to transfer not only based on recipients’ availability or embryo morphology but also on genomic estimates. This technology can be implemented for a wide spectrum of applications in livestock. In this review, we discuss the use of embryo biopsy for genomic selection and share our experience with Gir and Girolando Brazilian breeding programs, as well as future goals for implementing it in Brazilian bovine *in vitro* embryo production practices.

## Introduction

Embryo biopsy involves manipulating embryos to extract samples. It is a well-established practice in veterinary medicine (Thibier & Nibart, 1995) with several applications. These include sex determination (Hasler et al., 2002), identification of specific genes (Hochman et al., 1996) and exome sequencing-based genotyping (Mullaart & Wells, 2018).

Significant progress has been made in molecular biology techniques and equipment in recent decades, enabling the analysis of large amounts of molecular markers at a relatively low cost requiring lower DNA input in chips containing thousands of SNP (single nucleotide polymorphisms) of interest (Boichard et al., 2012). Global genome amplification technologies have also evolved, making it possible to genotype individuals using single or few cells, such as embryonic ones (Saadi et al., 2014).

Combining assisted reproduction techniques in cattle with genomic selection confers sophistication and has the potential to increase efficiency of animal production. In vitro production of embryos can maximize the number of offspring from genetically superior animals, and the use of genomic estimates for embryo selection enables targeted animal production. Embryo genomic evaluation allows to estimate the productive potential of individuals since their embryonic stage and embryos with high added value can be frozen and traded in straws or transferred to recipients. Economic and environmental losses resulting from the production and breeding of low-value animals, such as males in herds intended for milk production, animals carrying genetic anomalies, and even animals with performance below the minimum acceptable for a particular group, can be avoided. This practice can save embryos recipients, as the breeder can choose which embryo will be transferred based on genomic breeding value and reduces the exchange of recipients between properties and the resulting logistical and sanitary problems.

Genomic selection was introduced in the milking Gir and Girolando breeding programs by a collaboration between Embrapa and breeders’ associations in 2017. Our current challenge now is to enable the genomic selection of embryos throw biopsy samples in these breeding programs, in which three main technological challenges were identified: (i) offering simple and effective protocols to enable the diffusion of biopsy technology to small laboratories, (ii) associating the optimized biopsy protocol to whole genome amplification and genotyping using appropriate SNP chips, and (iii) ensuring that the entire process can be applied to Gir and Girolando databases, validating genomic estimated breeding values by comparing them with the values of born animals. We discuss these topics below.

## Embryo biopsy: old methods, new applications

### Embryo biopsy applications during the past decades

**Figure 1:**
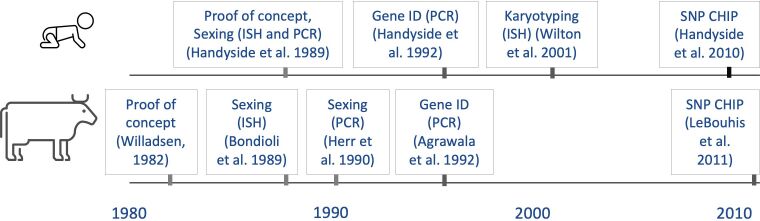
Use of embryo biopsy samples to perform genetic analysis in human and bovine - important milestones for genomic selection. Proof or concept: a sample from the embryo was biopsied and the embryo remained viable. Sexing (ISH): Y- chromosome identification by in situ hybridization. Karyotyping (ISH): Comparative genomic hybridization. Sexing (PCR): Y- chromosome specific genes identification by PCR. Gene ID (PCR): Specific genes identification by PCR. SNP CHIP: genome wide applications (exome sequencing).

The main reached milestones that paved the way throw genomic selection in cattle are summarized in Figure 1, compared to human medicine achievements. Biopsy sampling of pre-implantation embryos has been described since 1968 by Gardner (Gardner and Edwards, 1968), using rabbit embryos. Embryo biopsy has been used in cattle since the 1980s (Willadsen, 1982), initially to determine the sex of collected embryos before transfer to recipient cows throw conventional cytological methods (King, 1984). At this beginning, feasibility was a major concern: only 33 to 53% d7 embryos could be sexed using metaphase observation. The first milestone that paved the way to genomic selection in bovine embryos occurred when in situ hybridization was applied to identify the Y chromosome (reviewed by van Vliet et al., 1989), achieved first by Bondioli et al. 1989 (Bondioli et al., 1989). With the advent of PCR**,** biopsied samples from human (Handyside et al. 1989) and bovine embryos (Herr et al., 1990) were successfully sexed using this method, and analyses became more common in cattle (Hasler et al., 2002). This service has been commercially available in Brazil for more than 20 years**.**

The use of biopsied embryo samples for molecular marker assisted selection has been discussed since the 1990s, as genes of interest can be accurately identified in embryos for preventing genetic diseases (Handyside et al. 1992) or for selecting specific traits (Agrawala et al., 1992; Hochman et al., 1996; Saberivand and Outteridge, 1996). Then, with the advent of genome-wide SNPs panels for genotyping and selection of animals in the last decade, the discussion turned toward the application of genomic selection with SNP chips also for embryos (Humblot et al., 2010; Seidel, 2010), which is incomparably superior for genetic improvement than initial approaches when only a small group of genes was analyzed. Since 2011, this approach was proven possible (Le Bourhis et al., 2010). Currently, some commercial programs of European breeds already employ embryo biopsy and genotyping to select embryos with higher estimated breeding values for the production of the next generation of sires (Mullaart and Wells, 2018).

Embryo biopsies have another valuable application in studying the association between embryo profiles and outcomes. Biopsied samples can preserve markers from the original embryo, even after culture, and can be representative of the transferred embryo. This allows for the study of the relationship between genetic information and the success or failure of the embryo transfer, providing valuable insights for future reproductive strategies (Balvís et al., 2017; El-Sayed et al., 2006).

In the field of medicine, embryo biopsy for pre-implantation genetic testing (PGT) has been used since 1989 to identify the sex of embryos (Handyside et al., 1989) or specific genes (Handyside et al., 1993) and prevent genetic diseases (reviewed by Takeuchi, 2021). Advances in in situ hybridization technique allowed the karyotyping of biopsy samples and confirmation of euploidy, by comparative genomic hybridization (Wilton et al., 2001). Then, the development of karyomapping, a universal method for genome wide analysis was described for commercial purposes in human embryo biopsies (Handyside et al., 2010). Over the last decade, this technique has allowed the identification of genes and chromosomal alterations that may compromise the viability of embryos involving aneuploidies or chromosomal imbalances (Hayden, 2013; Yin et al., 2013). The association between aneuploid concepts and intellectual disability (Hassold and Hunt, 2001), and between couples with a history of reproductive problems and embryos with a high incidence of chromosomal alterations (Liang et al., 2013) supported the use of preimplantation genetic testing for aneuploidy (PGT-A) for human embryos. However, there is controversy since even though PGT-A is clinically recommended for aneuploidy screening, it was associated with a decreased cumulative live birth rate (Kucherov et al., 2023), leading recently to the editorial comment “PGT-A ‘perfect’ is the enemy of good” (Barad, 2023). Also, it has been demonstrated that human embryos have the ability of self-correction, by eliminating aneuploid cells (Orvieto et al., 2020). Therefore, recent data doubts PGT-A testing and suggests good embryos could be discarded based on false positive results.

In cattle, the first studies using karyotyping have estimated that 13.7% (Iwasaki and Nakahara, 1990) of in vitro-produced embryos present chromosomal aneuploidies (Viuff et al., 1999) demonstrated by in situ hybridization studies that this number is even higher because many bovine embryos (25% of in vivo-produced embryos and 72% of in vitro-produced embryos) are mixoploid, that is, mosaics that present aneuploid cells and normal cells. None of these studies was performed in embryo biopsies, aiming the development of commercial assays.

Recently, PGT-A human-like approaches have been applied to bovine embryos, such as the use of Karyomapping (a SNP-based screening test that allows detection of aneuploidy) (Griffin et al., 2019). Using SNP-based assays, 5% (Bouwman and Mullaart, 2023) to 14.1% (Silvestri et al., 2021) of chromosomal abnormalities, involving aneuploid and ploidy issues, were described. Aneuploid bovine embryos have only 5.8% chance of establishing a pregnancy after their transfer, compared to 59.6% chance of euploid embryos (Silvestri et al., 2021). An interesting study shows aneuploidy reflects on morphokinetics in bovine embryos, which might be used to build individual transfer decisions (Angel-Velez et al., 2023).

An approach that should be mentioned is the use of cell-free DNA from embryo culture medium for analysis. In 2016, it was proven that DNA released by human blastocysts into the culture medium (cfDNA - cell-free DNA) is useful for estimating the chromosomal content of embryos, with a high correlation with data from trophectoderm (TE) biopsy analysis to detect aneuploidy (Shamonki et al., 2016). A recent broader study showed that, despite different laboratory culture conditions, the concordance rate between cfDNA and TE biopsy was 78.2% (866/1108) (Rubio et al., 2012). Although TE biopsy is still considered the gold standard test for detecting embryo chromosomal anomalies, cfDNA analysis is promising for human embryo selection mainly because of its simplicity (Navarro-Sánchez et al., 2022). However, more studies are needed to understand the origin of cfDNA (epiblast or TE) and the mechanisms involved (Rubio et al., 2020). Additionally, the technique requires a specific protocol to be validated in each laboratory individually.

### Technical aspects of embryo biopsy

**Figure 2:**
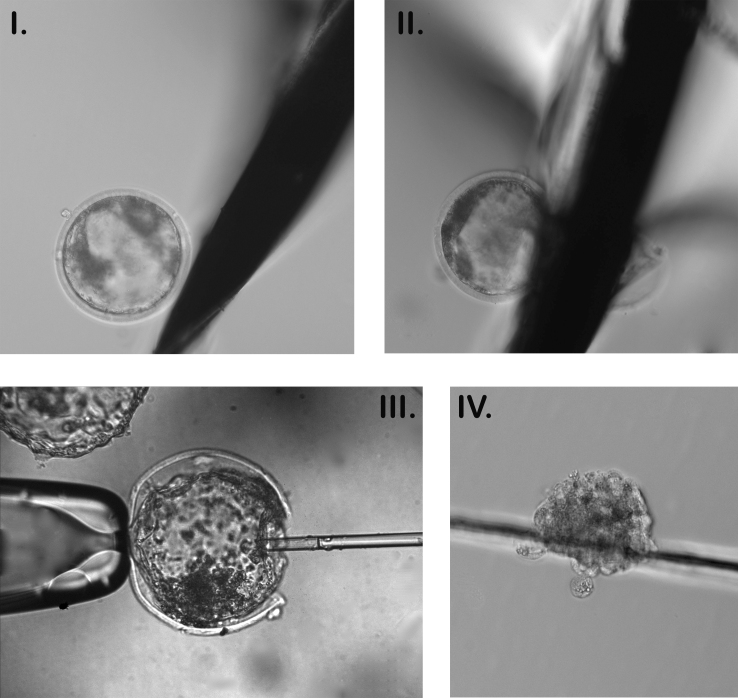
Biopsy of bovine blastocyst embryos. I. Blastocyst just before biopsy procedure, with micro blade next to it. II. Blastocyst is cut using the micro blade, avoiding the inner cell mass, to separate cells for biopsy. III. A blastocyst is positioned for micromanipulation biopsy, just before aspiration of cells. IV. An embryo biopsy sample is shown.

Performing biopsies on blastocyst-stage embryos has many advantages over other approaches described for earlier stage embryos (Griffin and Ogur, 2018), including the fact that at the blastocyst stage, more cells and a higher proportion of competent embryos are found. In this article, we will focus on the blastocyst stage, when the biopsy aims to collect around 10-20 trophectoderm cells. There are two main strategies described, exemplified in Figure 2, with minor adaptations for each. The first strategy involves using a micromanipulator, which stabilizes the blastocyst position with a holding pipette, and a micropipette for cell aspiration (Cenariu et al., 2012). There are several micromanipulators available, ranging from portable small ones to sophisticated equipment. Portable equipment has the advantage of being able to serve several IVF laboratories as an embryo biopsy service only. In the micromanipulation strategy, lasers can be used to improve biopsy removal by cutting the final attached cells, but this is an optional procedure mainly suited for laboratories that will perform a large number of biopsies. Laser-assisted biopsy in bovine has been reported (Tutt et al., 2020).

Another possible approach is the use of a microblade, also known as a handmade biopsy (Camargo et al., 2018; Peippo et al., 2007). In this technique, an ultrasharp blade, similar to the one used for embryo bipartition, is used to dissect a portion of the blastocyst for analysis. Although this technique allows almost every lab to perform embryo biopsies since it is simple, cheap, and does not require more equipment than IVF practice does, it has a decreased embryo survival expectation after the procedure (Ogata et al., 2015), and 20-30% losses are expected (compared to 10% in micromanipulation biopsy) based on our experience. In our experiments using handmade biopsy, no effect on pregnancy rates was present (Oliveira et al., 2017a). However, a comparison between microblade biopsy with micromanipulation biopsy revealed and lower pregnancy rates from frozen-thawed embryos when a microblade was used (Cenariu et al., 2012). In fact, microblade biopsy can be influenced by the microblade type and the blastocyst size, which was not considered in this study.

Several studies confirm the safety of biopsy for bovine embryos derived from both superovulation and in vitro fertilization. Although some embryos may be lost during the biopsy, those that survive the procedure and reexpand have good pregnancy rates (26.37 to 71%), equivalent to non-biopsied embryos in each paper (Carbonneau et al., 1997; Sousa et al., 2017; Humblot et al., 2010; Lopes et al., 2001; Oliveira et al., 2017a; Shea, 1999).

Embryo biopsy does not affect cryosurvival. Many studies in mouse and human embryos have demonstrated similar results, and biopsy coupled with vitrification has been followed in medical practice aiming to perform PGT before embryo transfer in human patients (Griffin and Ogur, 2018) Studies with bovine embryos show similar results, with no effect of biopsy on cryosurvival (González-Rodríguez et al., 2022; Najafzadeh et al., 2021; Takahashi et al., 2013; Tominaga, 2004), even though cryopreservation generally decreased pregnancy rates in bovine embryos, especially observed in earlier studies (Tominaga, 2004). Since cryopreservation protocols have been largely updated in bovine embryology during the past decades, storage of frozen-biopsied embryo while genotyping is performed, and breeding value is calculated is a good choice. For embryos produced by in vitro fertilization (IVF), the best outcomes after cryopreservation are typically achieved through the technique of vitrification, the preferred method in human medicine due to its proven minimal harm to embryos and superior results compared to slow freezing (Li et al., 2014; Rienzi et al., 2017). As expected, vitrification resulted in fewer apoptotic cells after thawing (Najafzadeh et al., 2021), what can be relevant for an embryo with a number of cells reduced by the biopsy, and suggests it may be recommended over slow freezing for biopsied embryos. One aspect that should be noted is that embryo biopsy compromises the integrity of the zona pellucida (ZP), and the international commercialization of embryos without the ZP is discouraged in most countries.

The size of the biopsy, the embryo stage, and the embryo quality can impact the survival of the embryo and the quality of the sample obtained for genotyping. These are important points to consider when performing embryo biopsy for genotyping.

Regarding the size of the biopsy, it is important to strike a balance between the risks to embryo implantation and the quality of the sample obtained. With fewer cells, the sample could be more sensitive to errors during the pre-amplification process. Missing genotypes and allele drop out drastically increased when less than 30 cells are processed throw MDA-based techniques, but embryo viability is reduced when more than 10 cells are collected throw biopsy (Lauri et al., 2013). Call rate, a useful quality control parameter for genotyping, is related to the size of biopsies. An increase from 0.78 to 0.94 was observed from one-cell biopsies to bisected blastocysts (Mullaart and Wells, 2018), while an increase from 0.91 to 0.98 was observed from 5-cell biopsies to 15-cell biopsies (FUJII et al., 2019). Hence, in our experience, we aim for biopsies with around 10-20 cells.

### Embryo stage and quality can affect survival after biopsy

The embryonic stage of blastocysts at the time of biopsy may affect the efficiency of the process, particularly for microblade biopsies. Early, regular, and expanded blastocysts have similar re-expansion rates, but after embryo transfer, pregnancy rates are increased in blastocysts that were expanded at the biopsy procedure (Oliveira et al., 2017a).

We noticed that expanded blastocysts with larger blastocoel cavities are easier to manipulate, resulting in a shorter procedure time and potential benefits for the structures. In early blastocysts, which were predominant on day 6 (46.1% under our conditions), it can be difficult to preserve the inner cell mass and remove cells due to their small size. Additionally, a reduction in the total number of cells is expected in biopsied embryos compared to controls (approximately 25% fewer cells in our conditions), reflecting the removal of cells for the biopsy sample and damage to surrounding cells. In this sense, an increase in apoptosis was also reported, which indicates the injury caused by the procedure possibly due to the microblade contact (Quintão et al., 2016). Following embryo transfer, the embryo undergoes hatching and initiates a process of extensive trophoblastic elongation, resulting in over a 200-fold increase in size (Assis et al., 2010). This process plays a critical role in establishing pregnancy (reviewed by Wiltbank et al., 2016). Since smaller embryos will probably have fewer trophectoderm cells after biopsy, this could have consequences for trophoblast elongation following embryo transfer, which is critical for pregnancy establishment (Wiltbank et al., 2016).

Embryo quality, as usual, affects pregnancy establishment in biopsied embryos, and grade 1 rates are higher than grade 2 (Bredbacka, 1998; Thibier and Nibart, 1995).

Another interesting finding concerns pregnancy losses. In a controlled study carried out at an Embrapa Dairy Cattle Experimental Farm, following transfer of non-biopsied and microblade biopsied embryos to recipients, we observed absence of pregnancy losses in biopsied embryos during the first trimester, unlike the control embryos (non-biopsied), which presented relevant losses (17.6%) (Oliveira et al., 2017a). Early embryonic losses with in vitro fertilized embryos are an expected phenomenon in cattle, in percentages of up to 25% (Thompson and Peterson, 2000), for the elimination of embryos with problems and incompatible with subsequent development. Possibly, since microblade embryo biopsy represents an injury, it may have selected more competent embryos and eliminated less competent embryos, which were not able to reorganize and re-expand after the procedure. This phenomenon is positive for the market, as it prevents expenses and mobilization of recipients and resources with embryos whose fate is pregnancy loss. The other parameters of the obtained pregnancies were normal and did not differ from the control embryos (non-biopsied), such as the pregnancy rate at 30 and 60 days, the birth rate, the duration of gestation, and the weight of the born calves.

### Biopsy processing for genomic selection

**Figure 3:**
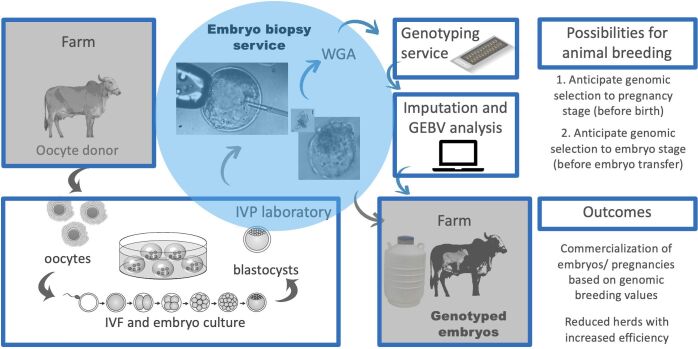
Biopsy application in bovine IVP. Oocyte donors are selected and their oocytes are recovered in the farm. Oocytes are taken to the IVF laboratory, matured, fertilized and embryos are cultured until the blastocyst stage. Embryo biopsy can be performed at this stage, by a third part. Following blastocoel reorganization, embryos can be destined for transfer or cryopreservation, and the biopsy sample is stored until whole-genome amplification. Amplified samples are sent to genotyping and results are analyzed after imputation. Genomic estimated breeding values are calculated and frozen or recipient’s embryos can be selected based on this information. IVP (in vitro production); IVF (in vitro fertilization); GEBV (genomic estimated breeding value).

The main steps for genomic selection using embryo biopsies are summarized in Figure 3.

## Whole genome amplification

Small samples have a limited amount of DNA template. A biopsy of 10-20 cells contains around 100 pg of DNA, while at least 200 ng of DNA is required for genotyping assays. Therefore, to perform genotyping from small samples, it is necessary to expand the genomic content in order to obtain sufficient DNA for commercial SNP panels.

Global genome pre-amplification in embryonic samples was initially described by (Chrenek et al., 2001), and its application is consolidated for pre-implantation embryonic diagnosis in panels composed of SNP markers (Barker et al., 2004). Thus, low initial genomic DNA input (including those obtained from a single cell, estimated at approximately 5-6 pg of DNA) can be expanded to micrograms of DNA (Zheng et al., 2011). In a previous study, our group had already verified that whole embryonic genome amplification procedures can be applied to bovine embryos, allowing the analysis of specific genome fragments (Polisseni et al., 2010). Various methodologies have been proposed to amplification, and commercial kits are available, the choice of which can affect the final results of genotyping (Treff et al., 2011). Currently, MDA-based technology is considered the most complete, returning superior genomic coverage results from bovine embryonic biopsy samples when compared to SPIA- and LMA-based technologies (Saadi et al., 2014).

In MDA method, random primers that cover the genome are employed to initiate DNA replication by polymerase. In this method, Phi29 enzyme has a displacement activity, unwinding the double helix and allowing additional primers and enzymes to access DNA at the site (Dean et al., 2002). The result is an exponential isothermal amplification of the template. After amplifying bovine embryo samples with this method, the material can be used for genotyping of SNP markers with satisfactory results (Le Bourhis et al., 2011; Lauri et al., 2013; Oliveira et al., 2023).

We conducted an experiment using Gir and Girolando embryo biopsies to evaluate the quality parameters of the MDA-amplified sample before sending it for genotyping (Martins et al., 2020). We found high variability in the results among the processed samples for spectrophotometer quantification and microfluidic chip electrophoresis analysis, and we could not identify any reliable parameter that could indicate the quality of genotyping before its execution related to quantity of DNA, integrity, and size of the fragments obtained after global genome amplification. Therefore, all biopsied samples are sent for genotyping in the current workflow.

Another point worth mentioning is the CallRate parameter (CR), obtained in return for the genotyping performed. Although very high in the case of genomic DNA samples obtained from hair or blood, this parameter is frequently reduced in biopsy samples submitted to global genome amplification. This phenomenon originates in missing genome regions, which were not properly copied in the amplification process and will be absent in the genotyping analysis, causing inconsistencies between the embryo's and the born animal's genotype (average percentage of inconsistencies of 1.6%, and the average percentage of SNP loss of 11.4% (Sargolzaei, 2013).

## Bovine SNP chips and imputation

Single nucleotide polymorphism (SNP) chips were developed as a high-throughput genotyping platform to genotype hundreds of thousands of SNPs across the genome. SNP chips have revolutionized the field of genomics, allowing for high-throughput genotyping of large numbers of samples at a relatively low cost. They are widely used in animal and plant breeding programs, as well as in human genetics research, and have led to significant advances in our understanding of the genetic basis of complex traits.

The development of SNP chips began in the late 1990s and early 2000s when the first SNP maps were being constructed for the human genome. The first SNP chip for cattle was the Illumina BovineSNP50 BeadChip, launched in 2008, which contained approximately 54,000 SNPs selected from a pool of over 800,000 SNPs identified in the cattle genome. The chip was designed to capture genetic variation across all major beef and dairy cattle breeds, as well as other populations of cattle. The BovineSNP50 BeadChip has been widely adopted by the cattle industry and is currently used in many cattle breeding programs around the world. Illumina has released several updated versions of the chip, including the BovineHD BeadChip, which contains over 777,000 SNPs, and the BovineLD BeadChip, which contains approximately 6,000 SNPs that are highly informative for linkage disequilibrium-based analyses. These SNP chips have greatly facilitated genomic selection and other applications of genomics in cattle breeding programs.

Whole-genome amplification inevitably results in a random loss of information and introduces erroneous genotypes, which poses a challenge for interpreting genomic results obtained from embryonic samples. Therefore, the accuracy of genomic information heavily relies on genotype imputation, a process where unobserved genotypes can be statistically inferred using known haplotypes in a given population. This approach increases the density of single nucleotide polymorphisms (SNPs) and expands the coverage of genotyping results (Oliveira et al., 2017b). By predicting the genotypes at the unassayed SNPs in the study sample, imputation enhances the number of evaluated SNPs, which can improve the accuracy of the genomic information (Marchini and Howie, 2010). Notably, imputation can correct more than 95% of errors in genotyped samples obtained from biopsied embryos (Saadi et al., 2014).

Some commercial breeding companies discard genotype results with call rates below 0.85 to minimize error rates in SNP genotyping and genomic estimated breeding values in European breeds (Mullaart and Wells, 2018). From our experience, no recommendation for call rate thresholds in Gir can be set yet, since this should consider the specific results obtained in each database. We observed a stable correlation of GEBV between embryo and calf samples across different classes of call rate samples when using Bovine HD imputation in Gir embryo biopsies (Oliveira et al., 2023). A closer association between the imputed embryo samples and the reference Gir population can positively affect imputation accuracy, as is observed for imputation of young Gir bulls data (Boison et al., 2017).

As reported (Hozé et al., 2014), imputation to high-density panels can yield higher reliabilities than imputation to low-density panels. Imputation to a high-density panel such as BovineHD allows for the use of information from most SNPs detected in lower density panels. Our group conducted a study using Gir embryo biopsy genotyping with the Z-Chip panel, and found that an increased accuracy of GEBV estimates and correlation to calf samples for Bovine HD imputation compared to imputation to the Z-Chip panel (Oliveira et al., 2023).

To further improve GEBV estimates in Gir embryo samples, using genotyping panels with closer association to the BovineHD panel would be beneficial, rather than relying on the Z-Chip panel. Additionally, the use of lower density panels for genotyping following imputation to high-density panels could be a cost-effective alternative for commercial applications.

## Use of genomic selection in brazilian breeding programs for Gir and Girolando Breeds

Genomic selection is based on the use of a high number of molecular markers spread all over the genome, together with phenotype data obtained from a reference population, to predict the phenotypic values of a non-phenotyped population. The estimation of genomic breeding values rapidly increases the genetic gain by shortening the generation interval in a given population (Meuwissen et al., 2001). With the decrease in genotyping costs, this technology has become a standard tool to assist cattle breeding in the northern hemisphere, pioneered with the Holstein breed in 2011.

The breeding programs for most dairy and beef breeds in Brazil take genomic information into account for genetic evaluation. Differences among these programs include parameters such as the number of genotyped animals, marker density, and evaluation models. In vitro embryo production is a consolidated tool for disseminating desirable genetics in genetic improvement programs. Therefore, nondestructive biopsy for embryo selection would be the fastest way to achieve the goals set in genetic improvement programs, reducing the generation interval and costs related to the milk industry (Chrenek et al., 2001).

Over the past 35 years, our team at Embrapa Dairy Cattle, in coordination with breeders' associations (ABCGIL and Girolando), has implemented genetic improvement programs for the Gir and Girolando breeds, resulting in a doubling of milk yield. In 2017, the implementation of genomic selection, for milk production and age at first calving traits in these breeding programs, has led to a significant increase in the genetic gain by reducing the generation interval through the selection of young sires and dams.

By 2018, genomic selection was definitively implemented into the Gir breeding program, resulting in the publication of the first genomic progeny testing summary for sires and dams. Since then, the number of genotypes, phenotypes, and pedigree information has significantly increased.

Genomic selection has also been used in the Girolando breed to correct pedigree errors and estimate the genomic estimated breeding values (GEBVs) of young sire candidates for progeny testing since 2016. Clarifide® Girolando, a commercial genotyping tool, was developed in partnership with Embrapa Dairy Cattle, the Girolando breeders’ association, Zoetis, and CRV Lagoa. It allows for the estimation of Genomic Prediction Transmitting Ability (GPTAs) for milk production, calving interval, age at first calving, and genetic tests for hereditary diseases in dairy cattle. Monthly analyses are performed and results of progeny testing with genomic evaluation for sires and dams are published once or twice a year in the herd book.

With genomic prediction tool, animals are selected before their milk production is verified with high accuracy, according to breeders' requirements. As a result, the demand for genetically superior animals, semen, and embryos has increased both domestically and internationally.

## Impacts of genomic selection in embryos for brazilian dairy cattle

The use of embryo biopsy in Gir and Girolando animals in Brazil is expected to be introduced gradually, starting with a small proportion of the animals that would be genotyped. Currently, this encompasses 100% of the Gir and Girolando bulls used in the National breeding program and 78% of registered Gir females in 2022. The Gir female percentage appears to be on the rise when compared to preceding years (74% in 2021 and 69% in 2020) (Silva, MVGB; personal communication). Furthermore, Brazilian breeders have demonstrated a strong inclination towards embracing technological advancements in the field of animal reproduction. This is evident from the significant increase in IVF adoption following its implementation in Brazil. According to statistics provided by ABCZ (ABCZ, 2023), the ratio of IVF-transferred embryos to total registered births in the Gir breed was 0.16 in 2005 (1763/10919), which increased to 0.69 in 2015 (12572/18259) and further escalated to 1.59 in 2022 (19847/12485).

The main benefit of embryo biopsy in cattle is genomic selection before the calf birth. Differences of up to 875kg of milk per lactation are found between sister embryos (Mullaart and Wells, 2018). In Gir, we observed a difference of approximately 800 kg of milk per lactation between the minimum and maximum GEBV embryos on the same property (Oliveira et al., 2023). Therefore, only desirable individuals would be produced. Embryos below farmer’s threshold could be sold to properties with less advanced breeding programs, or those focused on other traits. Male embryos and male calf management (undesirable for properties focused on milk production) could also be avoided with this approach.

Expenses related to the production of animals that will be destined for disposal could be avoided. The Brazilian cost of calf production was estimated at R$1,561 in 2017 (Gonçalves et al., 2017), and from birth until the first calving (moment when milk production begins), it was estimated at R$2,857 in 2014 (Santos and Lopes, 2014). Correction of these values to December 2022 using Embrapa’s ICP Leite (Milk Production Cost Index) returned R$3,357 for calf production cost and R$6,143 from birth to first calving. In the case of breeds that exhibit late puberty, such as the Gir breed, the cost may be even higher. Therefore, the employment of embryo biopsy and genomic selection before transfer is a low cost compared to the values discussed above.

Embryo genomic selection can also have an impact on the in vitro production market embryos in Brazil, which was about 441,000 embryos produced and 435,000 embryos transferred in 2021 (Viana, 2022). It can aggregate value to the service offered by IVP commercial companies and to embryos commercialized by the breeders as well. Embryos can be selected and negotiated accordingly to the demand of a specific region, farm or herd.

Genomic selection before embryo transfer also represents an important step towards sustainability, as the increase in animal performance and directed production preserves environmental resources, using smaller and more productive lands in the activity. Furthermore, a smaller and more productive herd is expected to have a lower carbon footprint, as the number of animals is directly related to greenhouse gas emissions.

## Conclusion

Due to the potential gains conferred by genetic improvement programs and dairy cattle industry, as well as the Brazilian characteristics of worldwide leadership in the in vitro embryo production market, and in the exploitation of Gir and Girolando breeds and their high adaptability to dairy production systems in the tropics, we believe that genomic selection in embryos will be part of the Brazilian reproductive biotechniques portfolio in the near future. Thus, this technology may catalyze productivity increases in Brazil, with a consequent reduction in the carbon footprint of milk produced. The use of DNA recovered from media represents a promising approach in this context, if it is proven compatible with bovine IVP and genomic selection protocols.
